# Cognitive-electrophysiological indices of attentional and inhibitory processing in adults with ADHD: familial effects

**DOI:** 10.1186/1744-9081-7-26

**Published:** 2011-07-13

**Authors:** Gráinne McLoughlin, Philip Asherson, Bjoern Albrecht, Tobias Banaschewski, Aribert Rothenberger, Daniel Brandeis, Jonna Kuntsi

**Affiliations:** 1 MRC Social, Genetic and Developmental Psychiatry Centre, Institute of Psychiatry, King's College London, De Crespigny Park, London SE5 8AF, UK; 2 Child and Adolescent Psychiatry, University of Göttingen, Von-Siebold Str. 5, 37075, Göttingen, Germany; 3 Department of Child and Adolescent Psychiatry and Psychotherapy; Central Institute of Mental Health; Square J5, 68159 Mannheim, Germany; 4 Child and Adolescent Psychiatry and Center for Integrative Human Physiology, University of Zürich, Neumünsterallee 9, 8032 Zurich, Switzerland

## Abstract

**Background:**

Attention deficit hyperactivity disorder (ADHD) is a common neurodevelopmental disorder that starts in childhood and frequently persists in adults. In a comparison of adults with ADHD and a matched control sample, we previously showed that abnormal inhibitory processing is typically preceded or accompanied by other processing deficits in adult ADHD. We now compare these data further to additional data from first-degree relatives (fathers) of children with ADHD to identify whether this pattern of abnormal processing shares familial influences with ADHD in adults.

**Methods:**

Using a family design, we compared 20 fathers of children with the combined subtype of ADHD with 21 adults with ADHD combined subtype and 20 controls in event-related potential indices of preparatory states and subsequent response inhibition processing as elicited by a cued continuous performance task.

**Results:**

Fathers of children with ADHD exhibited significantly weaker orienting attention to cues and inhibitory processing than the controls but not the ADHD sample.

**Conclusions:**

These findings provide evidence for the familial association of attentional orienting and response inhibition processes with ADHD in adults and indicate a familial and neurobiological link between ADHD in children and adults.

## Background

Attention deficit hyperactivity disorder (ADHD) is a common neurodevelopmental disorder that persists into adulthood in around 65% of cases and is associated with high levels of clinical, psychosocial and economic burden [1,2]. ADHD in adults is recognised as a valid and reliable disorder that is a developmental outcome of ADHD in children and shares many of the same clinical features with ADHD in childhood, including the cardinal symptoms of inattentiveness, overactivity and impulsivity [3-5]. ADHD tends to run in families with increased rates of ADHD among the siblings [6-8] and parents [6,9] of children with ADHD. Twin studies indicate that the familial risk for ADHD results from genetic influences with heritability estimates averaging around 76% during childhood and adolescence [10]. Due to the high heritability, aetiological investigations have focused primarily on the role of genetic factors and on the identification of intermediate phenotypes, such as cognitive and neurobiological processes, that potentially mediate genetic effects on behaviour [11-13].

Key requirements for intermediate phenotypes include association with the disorder, indicated by case-control differences, and presence in unaffected first-degree relatives of affected individuals with levels significantly higher than in the general population [14,15]. A limited number of studies have investigated event-related potential (ERP) indices as possible intermediate phenotypes in ADHD, with performance monitoring components emerging as promising measures of underlying processes that are sensitive to the condition and associated with ADHD both children and adults [16,17], although this may be task dependent [18].

The most established finding in the ERP literature on ADHD is that both children and adults consistently display deficits in motor preparation and attentional processes, which precede additional deficits in inhibitory processes [19-27]. In agreement with several studies on ADHD in children [20,21,25], we previously reported deficient covert attentional orienting and resource allocation, indexed by the P3 to cue stimuli, in adults with ADHD [28]. In the same study, reduced amplitudes of the contingent negative variation (CNV) component indicated further deficits related to the expectation of a stimulus, namely time processing, motor and non-motor preparation [28]. Again, these findings are in agreement with studies on ADHD in children [20,25,29]. Finally, our previous findings indicated abnormal inhibitory processing in adults with ADHD as indexed by an attenuated fronto-central P3 component to no-go stimuli [28], which again was similar to findings in children with ADHD [20,25]. The finding of attenuated P3 in adults with ADHD was also in agreement with studies in adults who had a childhood ADHD diagnosis [30], adults who scored above threshold on the ADHD symptom scales [31] and parents of children with ADHD [32].

The striking similarity of our previous findings on the cue P3, the no-go P3 and the CNV in adults with ADHD [28] to those previously identified in children with ADHD highlights the importance of these processes in relation to the ADHD diagnosis across the lifespan. This naturally suggests further investigation into the role of these processes in the aetiology of ADHD and whether they share genetic and environmental influences with those on the disorder. With this in mind, we have extended our previous investigation on case-control differences, by including an additional group of adults who are the fathers of children with an established diagnosis of combined subtype ADHD. If these processes share familial influences with ADHD in adults, we would expect the fathers of children with ADHD to be significantly different from controls in the specified ERP parameters.

## Method

### Sample

21 male adults with ADHD, 20 fathers of children with ADHD and 20 male healthy control adults participated in this study on the basis of informed consent. The joint South London and Maudsley and the Institute of Psychiatry NHS Research Ethics Committee approved the study (086/05). The age range was 18 to 56 years. A one-way ANOVA indicated a significant main effect of group on age (Table 1) with post-hoc analyses showing no significant difference between the ADHD cases and controls but significant differences between the ADHD cases and fathers and controls and fathers (Table 1). All participants had an IQ of 80 or above on the Wechsler Adult Intelligence Scale (WAIS-II) [33] and no main effect of group on IQ emerged (Table 1).

**Table 1:** Age, IQ and current and retrospective ADHD symptoms on the Barkley Adult ADHD rating scales

	ADHD (n = 21)	Fathers (n = 20)	Controls (n = 20)	ANOVA
Age, mean (SD)	32.51 (5.84)	45.90 (4.15)	30.00 (6.51)	F(1, 59) = 53.87, p < 0.001 A vs C:p = 0.48 A vs F: p < 0.001 C vs F: p < 0.001
IQ, mean (SD)	118 (10)	121 (13.37)	122 (12.10)	F(2, 58) = 0.67, p = 0.52
Current ADHD symptoms, mean (SD)	42.47 (7.62)	12.10 (8.81)	8.70 (8.30)	F(2, 54) = 81.90, p < 0.001 A vs C: p < 0.001 A vs F: p < 0.001 C vs F: p = 0.4
Retrospective ADHD symptoms, mean (SD)	22.00 (3.52)	7.90 (6.84)	5.90 (5.11)	F(2, 54) = 42.10, p < 0.001 A vs C: p < 0.001 A vs F: p < 0.001 C vs F: p = 0.5

All participants were right-handed, as determined by preferred writing hand, and had normal or corrected-to-normal vision. We previously reported the ADHD and control samples [28]. In brief, the adults with ADHD were recruited from the National Adult ADHD Clinic at the Maudsley Hospital, where they received the diagnosis from a specialist consultant psychiatrist, following a detailed clinical assessment to establish the DSM-IV criteria. Participants included in the study fulfilled criteria for DSM-IV combined subtype ADHD in childhood, and either combined type (n = 17) or inattentive type (n = 4) as adults. The adults with the inattentive subtype were just below threshold on the hyperactive-impulsive subscale (4-5 items). Exclusion criteria included the presence of an Axis I or Axis II co-morbid psychiatric diagnosis and taking any psychoactive medication other than stimulant medication for treatment of ADHD. A minimum of 48 hours medication-free period was required prior to the assessments.

The control participants were selected from a database of volunteers at the Institute of Psychiatry. They were selected if they had no major psychiatric conditions, substance abuse or previous head injury, and were matched with ADHD participants on age and gender.

The parent group was recruited from a database of families who had previously participated in the International Multicentre ADHD Genetics project (IMAGE). All of the fathers who participated in the current study had a biological child with a DSM-IV combined subtype ADHD diagnosis following a research diagnostic interview [28]. None of the fathers selected for this study had another psychiatric condition, history of substance abuse or previous head injury.

Self-report data were collected on current and retrospective ADHD symptoms, using the Barkley Adult ADHD rating scales [34] for all groups included in this study. Among the parent group, one father had a previous diagnosis of ADHD and scored above threshold on the rating scales; all other fathers scored below threshold. Among the controls, one participant had above-threshold symptoms for the inattentive subtype in the current ratings and two had symptoms sufficient to qualify for combined subtype from the retrospective not the current ratings; yet they had never sought treatment for their symptoms and did not consider themselves impaired. These individuals were not excluded from the main analyses, as the comparison samples were unselected for ADHD; the use of unselected samples enables unbiased estimates of the familial association between ADHD and secondary measures [35]. However, we separately examined the effect on results of excluding these four individuals. These additional analyses indicated that the four individuals were not outliers on any of the ERP or performance variables and excluding them from analyses did not change any of the results.

Fathers did not differ from controls in either current or retrospective symptoms whereas ADHD cases differed significantly from both fathers and controls in current and retrospective symptoms (Table 1).

### Procedure

Prior to the EEG assessment, participants' IQ was assessed using four subtests of the Wechsler Adult Intelligence Scale (WAIS-II): block design, vocabulary, picture completion and similarities [33]. The IQ assessment took 30 minutes in total. During the EEG assessment, participants were seated on an adjustable chair in an acoustically shielded, video-monitored room.

The task was a cued CPT with flankers [28,32,36]. This is a cued go/no-go task that probes attention, preparation and response inhibition or control, with incompatible flankers throughout to increase difficulty for adults [28]. Participants were instructed to respond only to cue-target (XOX-OXO) sequences by pressing a button as quickly as possible with the digit finger of their preferred hand. This instruction is assumed to cause a bias toward the go response, when the cue appears, so that stopping this prepared response requires increased inhibitory control or conflict monitoring. The task was practiced and comprehension ascertained prior to task performance. If necessary, participants were told to minimise eye movements or blinks. The task was run as part of a battery of three cognitive-electrophysiological tasks [28].

### Performance measures

Performance measures in the cued CPT flanker task included target reaction time (MRT, i.e. mean latency of responding in ms after target onset), within-subject variability in reaction times (SD-RT), and the coefficient of reaction time variability (CV, i.e. SD-RT/MRT), number of hits (target Xs detected between 200 and 1500 ms after stimulus onset), and number of false alarms (responses to letters other than target X). MRT and SD-RT were calculated across correctly answered target trials. Errors were broken down into subcategories (omission errors, total commission errors, and O-not-X commission errors).

### ERP recording and processing

The ERPs were recorded with a sample rate of 500 Hz and cut-off frequencies of 0.1-30 Hz via Nihon Kohden Ag/AgCl cup electrodes (impedances kept below 5 kOhm) fixed to the scalp with electrolyte gel at electrode positions, which included the 19 standard electrodes of the 10-20 system system plus Oz, Fpz and FCz (recording reference) using a Neuroscan recording system. Vertical and horizontal electrooculograms (EOGs) were simultaneously recorded from electrodes above and below the left eye and at the outer canthi. The EEG data were analysed using Brainvision Analyzer (Version 1.05) and, after down-sampling to 256 Hz, were corrected for horizontal and vertical (blinks) eye movements using the Gratton and Coles method [37]. Trials with remaining artifacts exceeding ± 100 μV in any channel were rejected from the digitally low-pass filtered (0.1 to 30 Hz, 24 dB/oct) data before averaging. All trials were inspected visually to detect additional subtle artifacts. Average ERPs were computed separately for each participant in three different stimulus conditions: (1) "go" trials (ERP to target OXOs preceded by a cue XOX), (2) "no-go" trials (ERPs to random letters following a cue XOX), and (3) cue trials (ERPs to a cue XOX). All averages were free from residual artifacts and contained a minimum of 20 accepted sweeps (Table 2). The ERPs were transformed to the average reference for all subsequent computations and topographical mapping. Calibrated zero baselines were used (instead of pre-stimulus baseline corrections) to avoid distorting the map topographies [38,39].

**Table 2:** Number of sweeps per stimulus, task and group

	CPT-OX with flankers		
	Controls	Fathers	ADHD
Cue, mean (SD)	75.30 (6.50)	72.15 (16.14)	75.94 (5.03)
Go, mean (SD)	37.90 (2.51)	35.70 (8.80)	35.71 (3.92)
No-go, mean (SD)	37.35 (3.90)	36.55 (8.26)	37.41 (3.32)

### Statistical analyses

**Figure 1:**
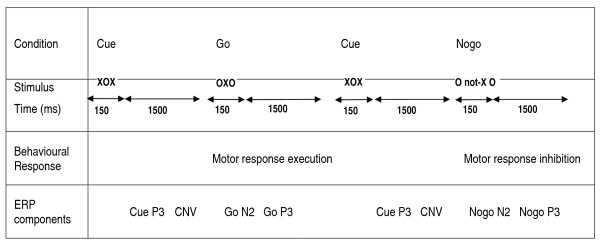
**Flanker CPT-OX paradigm**. The figure shows the relationship between task conditions and stimuli, behavioural responses and ERP components on cued go and nogo trials (10% each, 20% cues, 400 trials total). Note that the preparatory CNV following cues is sometimes also referred as a stimulus preceding negativity (SPN; preceding the go and nogo stimuli). See also Figure 1 in Banaschewski et al. 2004 for further detail.

Two ADHD participants were excluded from the ERP analyses due to excessive movements. ERP amplitudes were restricted to leads and time windows for which effects were expected to be largest, based on previous studies [40] (see Figure 1).

The *no-go-P3 *was calculated as the largest peak between 200 and 500 ms at Cz during "no-go" trials (random letters following a cue XOX). The *cue-P3 *was calculated as the largest peak between 200 and 500 ms at Pz during the presentation of cue (XOX) stimuli. The *go-P3 *was calculated as the largest peak between 200 and 500 ms at Pz during "go" trials (target OXOs preceded by a cue XOX). The *CNV *was calculated as the area at Cz between 1300 and 1650 ms during the presentation of cue (XOX) stimuli. The go N2 component was calculated as the largest peak between 150-300 ms at Fz during "go" trials. The no-go N2 component was calculated as the largest peak between 150-300 ms at Fz during "no-go" trials.

ERP latency data were analysed using analyses of variance (ANOVA). The significance of age as a covariate was tested on the ERP data using regression-based analysis and excluded from further analysis if non-significant [41]. Familial analyses on the amplitude of ERP components were conducted using nonparametric Kruskal-Wallis tests, with post-hoc analyses of the parent group versus other groups conducted using Mann-Whitney tests. The criterion for significant evidence of familiality employed here is a significant difference between fathers and controls.

Familial analyses on the performance measures were conducted using analyses of variance (ANOVA), with bonferroni analyses of post-hoc effects. As IQ did not differ between groups, we did not include it in analyses. As age significantly differed between the fathers and the other two groups, we initially included age as a covariate in familial analyses; we only report it where significant, as it was dropped from analyses otherwise. We adopted a significance level of p < 0.05 (two-tailed) throughout the analyses and, additionally, report trends (p ≤ .09).

Effect sizes (Cohen's d) for the ERP amplitudes were calculated using the difference in the means, divided by the pooled standard deviation of the raw data.

## Results

### Performance measures

Age was not significant as a covariate for any of the cognitive performance measures [all p > 0.51]. Fathers of children with ADHD were intermediate to ADHD participants and controls in all RT measures (Table 3) and main group effects emerged for these measures [all df = 2, 59; MRT: F = 9.29, p < 0.001; SD-RT: F = 8.18, p = 0.001; CV: F = 4.89, p = 0.01]. Post-hoc analyses indicated that ADHD participants had significantly longer MRT and increased CV and SD-RT than both healthy control and parent groups [all p < 0.01]. Fathers and healthy controls were not significantly different on any of these measures [all p > 0.65]. A main effect of group emerged for omission errors [H(2) = 12.09, p = 0.002] but not for total commission errors [H(2) = 0.27, p = 0.87] or O-not-X commission errors [H(2) = 0.15, p = 0.93]. Post-hoc analyses indicated that fathers made significantly less omission errors than ADHD participants errors [U = 105.50, p = 0.007] but not controls [U = 186.50, p = 0.91].

**Table 3:** Performance in the CPT-OX with flankers and effect sizes (Cohen's d) for group differences

	Controls*	Fathers	ADHD*	Cohen's d		
				Controls Vs Fathers	Controls Vs ADHD*	Fathers Vs ADHD
**MRT, mean (SD)**	377.76 (55.40)	390.21 (34.57)	468.79 (106.54)	0.27	*1.07*	*0.99*
**SD-RT, mean (SD)**	69.13 (36.18)	85.08 (44.92)	129.16 (62.21)	0.39	*1.18*	*0.81*
**CV, mean (SD)**	0.18 (0.07)	0.21 (0.10)	0.27 (0.09)	0.35	*1.12*	0.63
**Total commission errors**	1.0 (1.34)	0.79 (0.78)	2.19 (4.74)	0.19	0.02	0.41
**O-not-X commission errors**	0.45 (1.00)	0.32 (0.58)	0.57 (1.12)	0.16	0.11	0.28
**Omission errors**	0.45 (0.69)	0.79 (1.55)	2.76 (3.35)	0.28	*0.96*	*0.75*

MRT: mean reaction time in milliseconds

SD-RT: within-subject variability in RTs in milliseconds

CV: coefficient of variation (SD-RT/MRT)

* Previously reported in McLoughlin et al., 2010

Large effect sizes in italics.

### ERP parameters

Age was not significant as a covariate for any of the ERP measures [all p > 0.35]. The groups differed significantly in the latency of the cue-P3 [F(2, 59) = 3.88, p = 0.03] with the ADHD group activating the preparatory process earlier than the control group (p = 0.02) but not the fathers (p = 0.21). No differences emerged in latency between the fathers and controls (p = 1.00) (Tables 4 and 5). Analyses indicated a main effect of group for the latency of the inhibitory P3 [F(2, 57) = 3.36, p = 0.04], with a trend for differences between fathers and controls (p = 0.06); yet no other differences between groups emerged [ADHD versus fathers: p = 0.15; ADHD versus controls: p = 1.00].

**Table 4:** Mean amplitude (in μV) and latency (in ms) of components to cue, go and no-go stimuli in the CPT-OX with flankers for controls, adults with ADHD and parents (with standard deviation)

		Amplitude			Latency		
		Controls*	Fathers	ADHD*	Controls*	Fathers	ADHD*
**Cue**	**P3 (Pz)**	5.49 (1.92)	3.75 (1.58)	3.41 (1.63)	407.81 (67.84)	388.09 (65.54)	343.97 (95.41)
	**CNV (area at Cz)**	-3.72 (2.25)	-2.89 (1.77)	-1.88 (1.30)	#	#	#
**Go**	**P3 (Pz)**	7.71 (3.14)	5.31 (2.86)	6.90 (4.17)	364.65 (49.74)	404.08 (57.32)	374.13 (60.57)
	**N2 (Fz)**	-3.67 (2.39)	-3.26 (3.85)	-2.95 (3.14)	242.38 (33.01)	232.03 (63.08)	251.09 (41.96)
**No-go**	**P3 (Cz)**	7.56 (3.23)	5.16 (1.65)	4.57 (3.17)	366.99 (34.95)	399.41 (40.63)	373.05 (56.28)
	**N2 (Fz)**	-5.20 (2.97)	-3.67 (2.40)	-4.31 (2.57)	248.63 (26.86)	250.00 (34.59)	260.42 (25.98)

# There is no latency measure of the CNV

* Previously reported in McLoughlin et al., 2010.

**Table 5:** Effect sizes (Cohen's d) for group differences in mean amplitude (in μV) of components to cue, go and no-go stimuli in the CPT-OX with flankers

		Controls Vs Fathers	Controls Vs ADHD*	Fathers Vs ADHD
**Cue**	**P3 (Pz)**	*0.98*	*1. 17*	0.21
	**CNV (area at Cz)**	0.41	*1.00*	*0.65*
**Go**	**P3 (Pz)**	*0.79*	0.22	0.44
	**N2 (Fz)**	0.13	0.26	0.08
**No-go**	**P3 (Cz)**	*0.94*	*0.93*	0.23
	**N2 (Fz)**	0.57	0.32	0.26

* Previously reported in McLoughlin et al., 2010

Large effect sizes in italics.

**Figure 2:**
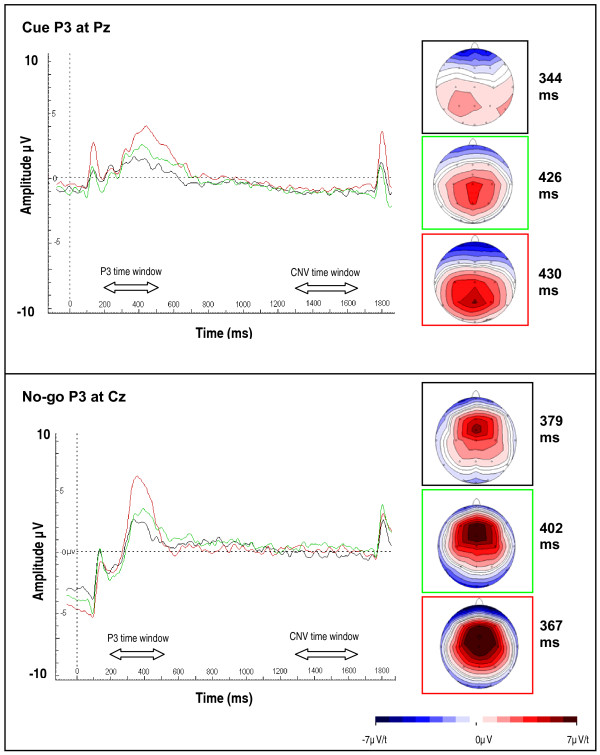
**Familial effects on cue and no-go P3 components**. Control participants in red, fathers in green and ADHD participants in black, with maps.

A significant main effect of group emerged for the amplitude of the cue P3 [H(2) = 12.91, p = 0.002] (Figure 2). Post-hoc analyses indicated that fathers significantly differed from controls [U = 92, p = 0.003] but not from the ADHD group [U = 152, p = 0.41] on the cue P3 amplitude at the Bonferroni corrected level of significance. Further, as reported in McLoughlin et al. (2010), a significant group difference emerged between ADHD and controls [U = 86, p = 0.004]. Similarly, a main group effect was evident on the amplitude of the no-go P3 [H(2) = 9.15, p = 0.01] and a significant difference emerged between fathers and controls [U = 9.15, p = 0.01], as well as between ADHD and control groups [U = 92, p = 0.01], but not between fathers and ADHD participants [U = 147.50, p = 0.34] (Figure 1). Analyses indicated a main group effect on the CNV amplitude [H(2) = 7.82, p = 0.02], but for this component fathers did not have a significantly attenuated amplitude in comparison to controls [U = 174, p = 0.50] whereas the difference between ADHD and control groups remained significant [U = 291, p = 0.004] and the difference between fathers and ADHD participants indicated a trend [U = 122, p = 0.06]. No main effects emerged for the go-N2 [H(2) = 1.61, p = 0.45], the no-go N2 [H(2) = 4.38, p = 011] or the go P3 [H(2) = 4.21, p = 0.12].

## Discussion

A comparison of 20 fathers of children with DSM-IV combined type ADHD, with 21 adult males with DSM-IV combined type as children and meeting criteria for ADHD as adults, and 20 healthy controls, indicates shared familial influences on ADHD in adults and attentional and inhibitory processes, as indexed by the cue P3 and no-go P3 respectively. Fathers of children with ADHD were significantly different from controls but were similar to a clinical sample of adults with ADHD in these two measures. Both genetic and shared environmental influences can contribute to the observed familiality of these measures. However, due to the relatively limited evidence for shared environmental influences on ADHD [42,43] and the cognitive and ERP measures reported here [44,45], it is likely that the familial association between ADHD and the cue and no-go P3 components index is explained by genetic factors. This should, however, be examined in future twin samples that allow for the estimation of genetic and environmental effects separately; as well as molecular genetic studies that aim to identify genetic variants that are associated with both ADHD and the candidate endophenotypes.

Despite a strong association between ADHD, in both children and adults, and response preparation, as indexed by the CNV [20,25,28,32], we did not find significant evidence for shared familial influences between this process and ADHD. The fathers were intermediate to ADHD participants and controls in the amplitude of this component, yet they were not significantly different from either group. This requires further investigation, to ensure that the lack of a significant finding here does not reflect inadequate statistical power. The moderate effect size, however, for parent and control differences in the CNV indicates that the familial effects on the CNV are not as strong as those for the cue P3 and the no-go P3. Further work in an extended sample is needed to clarify whether these represent state markers of ADHD in adults or trait markers of underlying genetic liability.

In terms of task performance, adults with ADHD were slower with more variable RTs than controls [28]. Fathers of children with ADHD were intermediate in these RT measures to ADHD participants and controls; however the difference between the parent and control groups was not significant. This is perhaps surprising since in studies on ADHD in children, one of the strongest findings of shared genetic/familial effects with ADHD for a cognitive variable is with RT variability [46-48]. In a recent large-scale ADHD and control sibling-pair study, a familial factor consisting of RT variability and mean RT accounted for 85% of the familial influences of ADHD [49]. Further work is now required to investigate the extent of these familial influences on ADHD in adults.

We also observed that the level of ADHD symptoms reported in the parents of children with ADHD was not significantly increased compared to the control group. Although this might seem surprising given the high familial risks for ADHD there are three potential reasons to explain this observation in our sample. First, the sample size is relatively small and may be underpowered to show a familial effect; secondly, although we did not control for unaffected status among the fathers who were invited to take part in this study, parents with high levels of ADHD symptoms may be less likely to participate in this kind of study; and finally, because self-rated ADHD symptoms in adults are known to show only small to moderate familial/genetic effects in twin studies [50]. The ERP group differences are particularly striking in light of these behavioural findings as it suggests that the ERP variables are more sensitive to the underlying familial liability for ADHD than behaviour itself.

In contrast, the significant familial effects for the cue P3 and no-go P3 show how sensitive these measures are to the underlying genetic risk for ADHD in adults compared to either the behavioural measures or cognitive performance measures. This suggests that the processes indexed by the cognitive-electrophysiological measures may be particularly important for our understanding of neurobiological processes that underlie risk for ADHD, but replication of these findings is required. Further work is therefore required to understand more about the aetiology of these sensitive markers of genetic risk and their relationship to the development of ADHD. A possible avenue for future research is the use of these measures in classification and discriminant analyses to identify if they are sensitive to genetic vulnerability for ADHD.

To test the generalisation of the findings reported here to more typical clinical samples, future studies could include individuals with common psychiatric comorbidities associated with ADHD, compare and contrast ADHD with the major co-morbid conditions and include a wider range of IQs. Another potential limitation in this study was the poor match for age between the fathers of children with ADHD and the ADHD case and control groups. The data do not, however, indicate an effect of age on any of the cognitive or electrophysiological variables used in this study. Further, if the cognitive processes associated with ADHD improve with age, reflecting the general tendency for reduced ADHD symptoms in adults as compared to children, we would expect any bias due to the age differences in the sample to lead to an overall reduction in father-control differences.

In conclusion, we obtained evidence for shared familial influences on ADHD in adults and attentional and inhibitory processes indexed by sensitive cognitive-electrophysiological measures. These processes may be particularly useful in studies that aim to further our understanding of the mechanisms that mediate genetic influences on hyperactive-impulsive and attentive behaviours. Further research should aim to clarify whether these represent pleiotropic effects of genes or whether these are intermediate phenotypes that mediate effects between genes and the developmental processes that underlie the onset and course of ADHD throughout the lifespan. Understanding the aetiological influences and interactions of the various processes involved may elucidate predictive indices for clinical outcomes in ADHD that can be used to target interventions and develop novel prevention and treatment strategies.

## Competing interests

The authors declare that they have no competing interests.

## Authors' contributions

GM carried out the EEG assessments, performed the data analysis and drafted the manuscript. BA, TB, DB and AR provided training and supervision in EEG analysis. PA and JK conceived of the study, and participated in its design and coordination and helped to draft the manuscript. All authors read and approved the final manuscript.
